# IoT Sensor Network for Wild-Animal Detection near Roads

**DOI:** 10.3390/s23218929

**Published:** 2023-11-02

**Authors:** Mindaugas Knyva, Darius Gailius, Gintautas Balčiūnas, Darius Pratašius, Pranas Kuzas, Aistė Kukanauskaitė

**Affiliations:** 1 Department of Electronics Engineering, Faculty of Electrical and Electronics, Kaunas University of Technology, LT-51368 Kaunas, Lithuania; pranas.kuzas@ktu.lt (P.K.); aiste.kukanauskaite11@gmail.com (A.K.); 2 Faculty of Electrical and Electronics, Metrology Institute, Kaunas University of Technology, LT-51368 Kaunas, Lithuania; darius.gailius@ktu.lt (D.G.); gintautas.balciunas@ktu.lt (G.B.); 3 MB “Dok inovacija”, LT-45252 Kaunas, Lithuania; pratasiusdarius@gmail.com

**Keywords:** wild-animal detection, IoT sensor network, thermo-vision camera

## Abstract

The monitoring and detection of wild animals is a significant topic for researchers who study the behavior, lifestyle, and environment of wild animals, as well as for people who encounter wildlife both in residential areas and near roads while traveling. An innovative wild-animal detection internet-of-things (IoT) sensor network running on harvested solar energy and detection methodology is described in this article. The sensor-networks node is implemented via the principle of an embedded system incorporating passive infrared sensors, a long-range (LoRa) module, and a solar panel for energy harvesting. For experimental purposes, a small IoT sensor network was implemented near the road. The network consists of eight nodes placed near the road with a distance of 50 m between nodes, a gateway for gathering detection data from the nodes, and a thermo-vision camera for verification of the received data.

## 1. Introduction

Animal-monitoring and detection methods have been used in wildlife migration observation. Animal behavior research is mainly aimed at the finding and tracing of the migration routes and determining their influence on the environment. The presence of wildlife in residential or farming areas and roads can be dangerous and is related to the risk of damage. The improvement of safety on the roads can be achieved by taking measures to prevent vehicle accidents that include wild animals [1,2]. For those aforementioned reasons, animal-monitoring and detection systems have been created. The detection systems work as a sensor network. They consist of sensors, wireless modules, microprocessors for data acquisition and processing-algorithm implementation, and warning devices for the dynamic transmission of warning signals [1,3,4]. The existence of such systems improves the lifestyles of people who live or travel near wildlife territories and helps researchers in zoology science as well [4,5,6].

Previously mentioned systems that consist of a few parts usually are composed of data collecting, data processing, and the indication of results or alarm parts, depending on the purpose of the system. Data are acquired by scanning the readings of various sensors as well as camera images; then, the records are transferred to processors where synthesis, processing, or artificial intelligence models’ algorithms are used for computation to extract useful results. If the system is intended to warn or provide information when the conditions for a particular signal rising are met, the information is conveyed via radiant signs and other means of communication. For a research-purpose, system information generally is transmitted wirelessly using BLE, WLAN, and other technologies depending on the distance between the interfacing devices and the requirements for the system itself.

In scientific publications that describe an examination or the creation of animal-monitoring and detection systems, beam-termination or coverage methods are common for the collection of information and data [1,2]. Beam-termination techniques include microwave, infrared, or laser signals [3,7]. Area-coverage methods process microwave, infrared signals, or camera images [2,4,5].

The principle of the operation of systems using the beam-termination method is that the transmitter broadcasts a signal to the receiver [3].

The model of a beam-termination system was described in a methodology proposed by Yusman et al. [7]. The research describes the design of a wild-animal detection and rescue system with passive infrared and ultrasonic sensors. The system detects animals when their bodies block the path of the signal or when the signal obtained by the receiver is greatly reduced, depending on the type of the signal. In these systems, the detection line is usually almost or completely linear; however, there may also be cases where the transmitter radiates the microwave signal at an angle or the signal transmission itself makes the detection line wider—the beam width of the transmitted signal is extended. The space between the transmitter and the receiver must be free, i.e., the field of view of both the transmitter and the receiver must not be obstructed by any objects, because this is the only way to ensure appropriate signal transmission. The principle of operation of area-coverage methodology is that the sensors which have a cone-shaped detection signal form are used. Animals are detected using the images received in real time from the cameras or sensors that detect movement and/or body heat [1].

A. Mukherjee et al., in the research described in [5], propose an area-coverage system that can detect large animals approaching the highway and accordingly alert the drivers of the upcoming danger. In the mentioned system, animals are detected in real time using microwave radars. Those types of systems are active area-covering systems that transmit a signal over the area under observation and process its reflection to detect animals. Passive area-coverage systems detect animals only from the received signal. Infrared and video cameras are the most popular choices. Algorithms for the data processing of systems using the beam-interruption method are simpler than those of the area-coverage type since the processed signals possess an ordinary form and the number of signals received by the sensors directly depends on the number of sensors. In systems using area-coverage methodologies, digital image processing and machine- and deep-learning algorithms are often used for data analysis, and the system can not only process data in real time and store it but also learn from those records [8,9].

Other types of systems can use cables buried underground; seismic, acoustic, magnetic, ultrasonic, and passive infrared sensors; and the methodology of systems worn on the body of an animal [10,11,12,13,14]. In cable-based systems, double-cable sensors of a certain length (several hundred meters) are dug underground and are used to detect large and medium-sized animals crossing the cable along its entire length [10].

C. Druta et al. propose the aforementioned cable-based system in their research [10]. The methodology can be used to detect large- and medium-sized animals and is implemented with 300 m of dual-cable dug underground. When the detection field created above the cables is disturbed, an alarm is set off, and it is determined where the animal has crossed the field. In sensor (ultrasonic, seismic, acoustic) systems, using data processing and merging algorithms, the combination of parts of the results are obtained from several data sensors which help to distinguish and recognize animals crossing/limiting the field of vision of the sensor [11].

T. Damarla et al. [11] propose in their research a solution for animal and personnel detection consisting of processing several sensors individually and evaluating the results of the analysis in a hierarchy. It is also possible to process several sensors individually (passive infrared, seismic sensors); the results from both sensors can be compared, and/or one sensor complements the other. For example, passive infrared sensors that passively receive infrared radiation are less sensitive to nonanimal environment changes than seismic sensors that are located on the ground [12]. Various types of sensors (movement, temperature, humidity, etc.) can be used in systems worn on animal bodies. The received data are processed and used to determine the location of the animal by sending signals wirelessly [15]. In addition, to increase the accuracy of such systems, the number of animals carrying the devices in a conventional area of their residence is periodically updated [14].

Using the beam-termination method, the maximum distance at which the system can detect an animal is generally much greater than that of the covered area systems; in reviewed studies, the systems based on the area-coverage method can detect the animal at a maximum distance of 61–165 m, while the beam-termination systems can detect the animal at 27–402 m maximum distance [1,3]. In beam-termination systems, it is impossible to specify the direction in which the animal is moving. Animal detection might be considered inadequate depending on what the system is used for because the system detects an animal that has crossed the signal beam, both when entering and leaving the monitored area. A system using such methodology must also be fixed particularly firmly because the beam of the signals used in it, and therefore the detection field of the system itself, is narrow, and, if affected by the environment provided it was not fixed properly, the equipment might issue incorrect data. Meanwhile, in area-coverage systems, it is possible to recognize the direction of animal movement by storing previously received signals and comparing them. In terms of improper mounting, the aforementioned system that uses passive infrared sensors, for example, because of wind, may simply become less sensitive, but finer sensibility does not have a significant impact on obtaining and processing appropriate data. As for unconventional, manifold types of systems, it is worth mentioning that, when using cables, such systems are invasive and require people or special equipment to lay the cables. By adding more sensors, although the probability of errors in detection is reduced, the analysis of the incoming data and interpretation of the results becomes more challenging. When placing collars on animals to detect their appearance in certain areas, it is necessary to arrange the required number of collars, as well as to install special stations that can receive the data broadcasted when the animal with a collage-type system approaches within a certain distance.

In more recent animal-monitoring systems, animal detection with cameras is becoming more popular, and various methods, models, digital image processing, and machine- and deep-learning algorithms are used for image processing [8,16,17,18,19]. The most common type of artificial neural networks used in systems processing images obtained by cameras is convolutional neural networks, which are used for image analysis [8,16,19]. In the photos submitted to the networks for animal recognition, specific characteristics of animals are discovered with the help of feature descriptors; thus, the artificial intelligence model is trained with a preprepared and labeled data set. Some of the used popular feature descriptors are the oriented gradient histogram, where features are recognized by normalizing the radiometric image and looking for the brightness and gradient of each pixel; a popular algorithm—a transformation of invariant scale features, which determines certain key points and provides them with quantitative information—assigns descriptors to them [16,17].

Another variant of feature descriptors is described by S. Matuska et al. in their research [17]; the authors propose an ASFAR system that includes operation on images from a “watching device” and calculation algorithms in the main computation unit. In the described methodology, a SURF Key Points Detector is used to find key points in the image, and SIFT, SURF, and opponent colors descriptors are used to describe key points by the n-dimensional feature vectors. The chosen methods and algorithms also depend on the field of use of the system in development. In a methodology proposed by Qiming Zhu et al. in their research [8], livestock monitoring is done via an image-processing algorithm based on shape-constraint classifiers and determining the weight and other characteristics of farmed animals based on predictable fixed dimensions of animal composition. Other systems, suited to monitor animals in the wild by studying their lifestyle and migration routes, use a network of cameras [19,20]. Taking into consideration the fact that animal monitoring requires minimal intervention in the animal’s habitat area and the minimum time spent in it for maintenance and/or repair of the equipment. One of the proposed methods by Z. Chu et al. in their research [19] is to use cameras that automatically trigger video capture, capturing images when animals appear in them, in this way reducing the number of images sent and increasing the camera’s lifetime. Another method described by Y. Nakayama [20] is to set the intervals at which images can be captured, as well as reducing the amount of data transmitted and keeping wildlife tracking efficient and stable, and this is done by using an age-of-information metric for evaluating the recency of data by measuring its delay from the perspective of the recipient.

IoT technology offers flexibility in terms of system configuration, network structure, sensors’ implementation, and performance optimization. IoT technology is recognizable in recent research works and mainly is being applied for animal monitoring. J. Arshad et al. in their research [21] present a smart dairy concept with attachable sensor nodes used for cattle coordinate tracking and live monitoring of body parameters, such as temperature, heart rate, etc. L. Nóbrega et al. in their publication [22] present the structure of the IoT network for livestock (particularly sheep)-monitoring collars to acquire the states of eating off the ground, standing, walking, running, etc.

The appearance of wildlife in urban areas is quite rare in Lithuania; the majority of vehicle accidents with wild animals are recorded on trunk roads at night. In Lithuania, from January to October 2023, 4764 accidents with wild animals were reported. This makes up 26% of all traffic accidents [23]. This percentage is constantly increasing, and it doubled since 2017. The population of wild ungulates in Lithuania is large and is increasing. About 19,000 moose, 68,000 deer, and 169,000 doe-deer populations were reported in 2022 by hunting associations. The wild boar population in Lithuania, according to data from 2022, is about 30,000 individuals [24]. Various solutions of modulations as OFDM/FDMA DBPSK (Sigfox) or LoRa [25,26], the technologies used in wireless LPWAN [27,28,29,30], enable the technical background diversity and usability in various scenarios for wireless animal tracking purposes. The published research works that are aimed at animal tracking utilize the concept of wireless network solutions. Bluetooth low energy (BLE) and LoRa are referred to by Ayele et al. in their publications [31,32]. The approach of wild-animal tracking in this scientific work is based on dual radio-network architecture to realize collar communication as the gateway for wildlife monitoring purposes. A similar approach to tracking wild animals with collars via a LoRa network is described by O’Kennedy et al. in their publication [33]. The tracking of individual animals is possible and applicable for small herds of big animals, but, in road safety applications, animals without collars cannot be tracked. The method of animal tracking using image data is proposed by Meenakshi et al. In their publication [34], they offer an optical range image analysis and recognition method within the LoRa network to implement animal recognition and classification between wild and domestic animals. This method is suitable for farming applications but requires high processing capabilities (Raspberry pi) for solution. In road safety applications, where most of the animal appearances on the road occur during the dark period of the day, this method is considered not prospective for the end product. A similar method was proposed by Bandari et al. in their publication [35] by classification of the optical range camera captured images by machine-learning algorithms and alerting via LoRa communication. It is very difficult to get the photographic quality of the images and also for the animal detection system to operate autonomously; the image capturing has to be activated during the presence of the animal to reduce energy and processing expenses, which was not addressed in works [34,35].

The image capturing is foreseen as an additional information source and the authors consider also thermal imaging assistance for the identification of wildlife movement in the dark period of the day. The overviewed methods [31,32,33,34,35] are considered to have drawbacks (due to the necessity of attaching collars to animals or processing optical range images). The concept of wildlife detection proposed by the authors of this paper is a low-power and autonomous stationary sensing network with visual signaling functionality. For quality of service, Sigfox and LoRa utilize unlicensed frequencies; NB-IoT uses LTE-licensed bands and can provide the same QoS as guaranteed by mobile operators. Regarding the reliable measurement devices’ data transfers, the NB-IoT is a better choice compared to LoRa or Sigfox.

As for battery life, NB-IoT [36] here loses to LoRa [36,37] and Sigfox [37,38] because it consumes additional energy needed for synchronous communications and QoS handling. In addition, OFDM/FDMA consumes more peak power than DBPSK (Sigfox) or LoRa [25,26].

Sigfox has the smallest data payload, only 12 bytes, and a limited message count per day. The LoRa payload is bigger, but it has restrictions for packet time on air. NB-IoT has the biggest payload and data rate [36].

In rural areas, theoretically, Sigfox is the choice because one base station can cover the biggest area, but Sigfox works only in some parts of the world [39]. In the city environment, Sigfox can cover up to 10 km, and LoRa can cover up to 5 km. The NB-IoT radius depends on the mobile network of a certain area, but it uses a well-developed mobile operators’ network and infrastructure.

The main advantage of the usage of LoRa for the wild-animal detection system is the possibility to use its own network at no cost per one device or one message sent.

So, in summary, each technology has its own advantages, but for the implementation of wild-animal tracking near the road system, LoRa is considered by the authors as a better choice.

Various wireless technologies can be used at LPWAN [27,28,29,30]. Table 1 shows a summary of the most used nowadays.

IoT technology, to the authors’ best knowledge, has not been widely reported to be applied to wild-animal detection systems, and the research aimed at this topic is considered to be relevant. The emerging long-range data-transmission energy-efficient technology (LoRa) can significantly extend the possibilities and flexibility of the system and increase the physical length of monitored roadway segments using a flexible-solutions network. The solutions may benefit from the decreased energy consumption using the low-power solutions and autonomic functioning of the nodes powered by renewable energy sources.

In this article, the IoT sensor-network structure and implementation that are presented can detect animals near roads in various weather conditions and seasons. The proposed animal detection system uses an IoT nodes network for the detection of wildlife near main roads. Nodes were mounted on roadside-marker posts and can detect wild-animal presence near the road. The nodes are designed to detect animal presence near the road and inform drivers using a flashing LED light. Each IoT node has a solar panel and runs on harvested solar energy. The testing system has a gateway with a thermal vision camera placed near the IoT node network. The thermal camera takes thermal photos of the road periodically. For research purposes, the gateway gathers raw node-activation data to be compared with the thermal photo footage.

## 2. Materials and Methods

### 2.1. IoT Detection System

The prototype animal detection system must be able to detect animals moving in the roadside area and inform the drivers of passing cars about them. The system consists of separate animal detection and signaling IoT nodes that have the possibility of mutual communication. For testing purposes, the system gateway and thermal vision camera were added.

**Figure 1:**
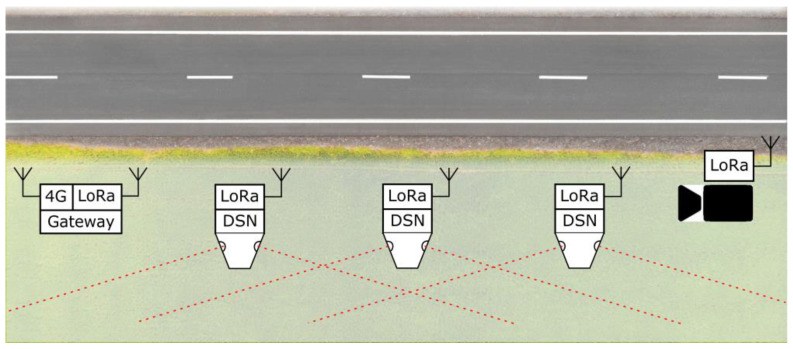
Animal detection IoT network with the possible placement of the DSN nodes.

The proposed detection-signaling nodes (DSNs) are installed on the roadside-marker posts (Figure 1). Roadside-marker posts, according to the standard, must be installed every 50 m on trunk and country roads. In the cases of straight sections of the roads, the distance between the marker posts can be increased to 100 m due to economical and technical reasons [40]. Roadside-marker posts are anchored to the roadside at least 0.5 m depth.

In order to ensure flexibility and to simplify the installation of the system on the road section and its expansion, the DSNs are designed to be fully autonomous. As an additional feature, low-power radio modules were provided in each DSN. Radio signals were used to communicate between DSNs to activate signaling beacons in several adjacent DSNs and thereby extend the warning-area distance. Figure 1 shows the animal detection IoT network with the possible placement of the DSN nodes near the road using already existing roadside-marker posts. The system can be made up of DSN units installed next to each other in a row (at max. 50 m). Sensitive areas of operation of adjacent DSN nodes overlap. As the animal moves toward the DSN nodes, several of them are likely to detect and start signaling. The network consists of several DSN nodes that detect animal presence near the road, a photo camera (CAM) that can be triggered using LoRa messages, and a gateway (GW) that logs triggering data received from the DSN. The gateway also can be accessed using a mobile 4G connection.

### 2.2. Structure of IoT Sensor Node

**Figure 2:**
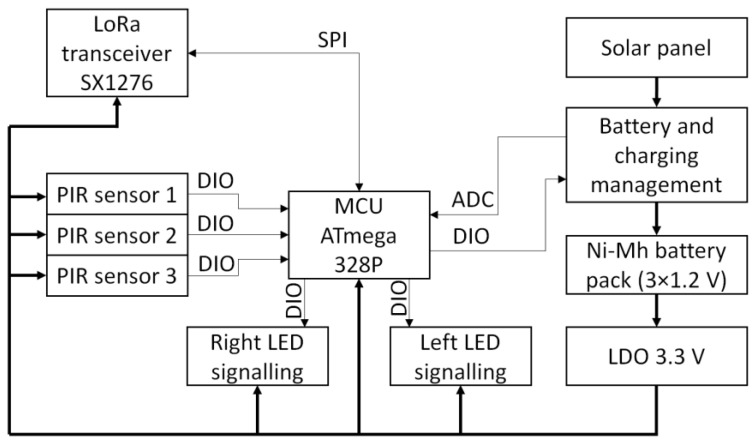
DSN internal structure.

Figure 2 shows the structure of the DSN. The node consists of a microcontroller (MCU), a radio communication module (LoRa transceiver), motion sensors (PIR—passive infrared sensor), and visual signaling elements (LED—light emitting diode). DSN is powered by batteries that are charged using a solar panel. A low-dropout regulator (LDO) provides 3.3 V to all components. When choosing the components of the DSN node, radio communication module, microcontroller, and motion sensors, the focus was on energy efficiency and price. Semtech SX1276 modules using LoRa modulation were selected for wireless communication between the Gateway and DSN nodes. These modules are characterized by low energy consumption when working in both sleep and active modes and long (up to 10 km) transmission distances [41]. When choosing a microcontroller, the power consumption in sleep mode, price, and availability in the market were considered. The selected Atmega 328 microcontroller, which consumes up to 10 μA in sleep mode, has enough digital input–output pins (DIO), a number of analog inputs (ADC), and an SPI interface (serial peripheral interface) for controlling the wireless communication module [42].

### 2.3. DSN Mounting

**Figure 3:**
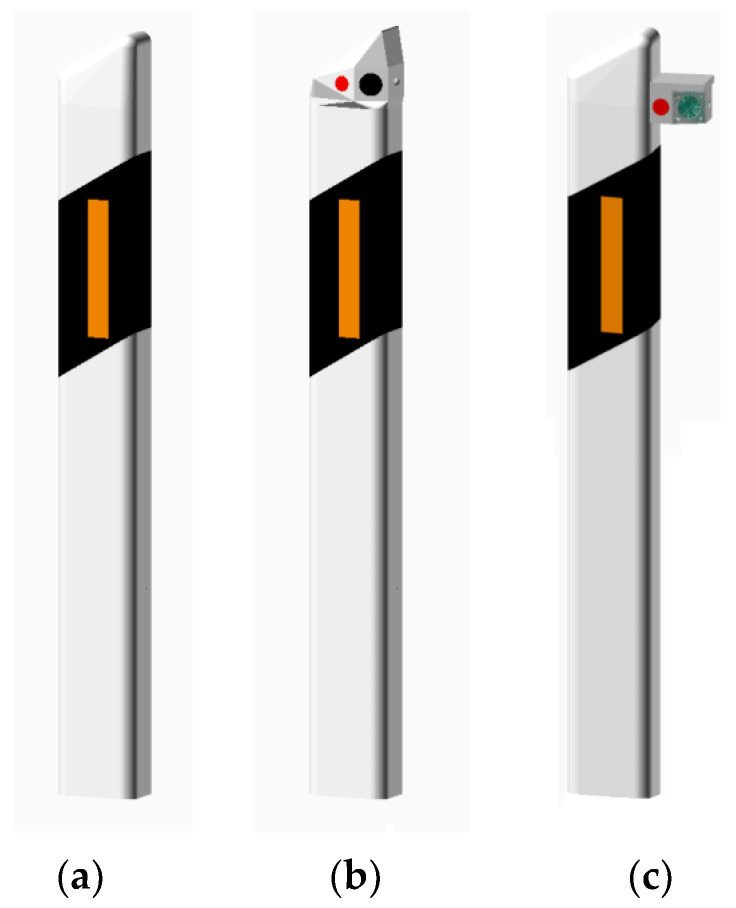
Realization options of DSN mounting on the roadside-marker post. (**a**) A standard roadside-marker post and two possible modifications: (**b**) With a modified 10 cm length, the upper part of the roadside-marker post with solar panel and (**c**) With an attachment mounted on the rear part of the roadside-marker post.

The existing road infrastructure was used for mounting the DSN units. Several mounting options were considered, but, for the realization of the sensor-network installation, the designs of DSNs that do not require mechanical intervention to the roadside-marker posts were chosen. Two mounting options are presented in Figure 3. The standard unmodified roadside-marker post is shown in Figure 3a. The DSN node shown in Figure 3b is mounted on top of a standard roadside-marker post. In this case, the DSN node is incorporated into the standard roadside-marker post without significant and visible design and dimension changes of the original roadside-marker post. The mounting variant from the side of the DSN node to a standard roadside-marker post is shown in Figure 3c. It has a smaller volume of the casing which results in better sealing, the smaller mass of the DSN, and is better protected from snow plowing during the winter period and water or snow splashing from passing trucks during rain or thaw. However, mechanical intervention to the roadside-marker post is necessary during installation (drilling or gluing), and the available area for mounting the solar panel is small. The solar panel can appear in the shadow of the roadside-marker post as well. The implementation of the case (Figure 3b) does not require a change in the dimensions of the roadside-marker post (width, depth, and height). The standard shape of the roadside-marker post is maintained and visual appearance changes from the driver seat are not noticeable. This design is used for DSNs of generation 1 and generation 2. The main advantage of the (b) mounting case is simple installation to the already existing roadside-marker posts. The solar panel is mounted on the top of the DSN module where a larger area for a solar panel is available, which results in better exposure to the Sun. The DSN node sensors can be blocked by grass and bushes, and the mechanical damage risk during grass mowing, snow plowing operations, or road construction works is higher.

**Figure 4:**
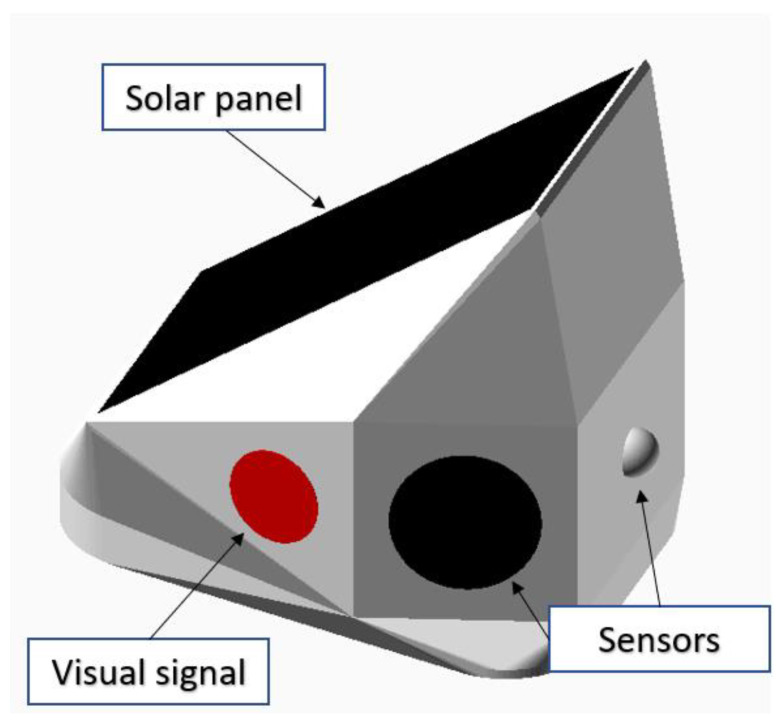
Visualization of the DSN node. Arrangement of functional elements.

The inclination angle of the solar panel in normal conditions is required for the best available average exposure angle to the Sun. However, in the case of the DSN installation, the inclination angle cannot be adapted to be oriented at the optimum horizontal angle. It mostly depends on the road section’s orientation. For example, the best solar energy capture would be ensured in the road section which has the direction from west to east with the solar panel oriented to south. The idea of the flat top was also considered for equalizing the solar energy capture for all available road orientations, but flattening the top of the DSN case would result in a smaller area for the solar panel, which is very important for better energy harvesting. The flat top would result in water and snow deposits, and the dust could not be washed by rain and would accumulate faster on the surface of the solar panel. Therefore, a vertical angle similar to the original was chosen which allows for the achievement of a visual appearance match by sacrificing some of the energy efficiency of the solar panel due to a nonoptimal vertical angle. The optimal electricity production occurs when the solar panel faces south at approximately the angle of the location’s latitude. In Lithuania, it would be approximately 54 degrees. The installed system can be used on both sides of the road. In this case, the performance of nonoptimally installed nodes could be partially backed up by those that are under better conditions, or the installation can be not performed for those road sections that appear in permanent shadow or the DSNs that have solar panels inclined to the north. Figure 4 shows a more detailed DSN 3D visualization. The body of the DSN assembly is 3D printed using white printing material. Three PIR sensors are mounted on the back walls of the DSN, facing from the road towards the forest. Two PIR sensors are mounted on the left and right sides of the DSN, with the sensors pointed along the road, and one aimed perpendicularly to the road direction, towards the forest (with lowered sensitivity).

Visual signaling elements are directed to the side of the road and installed on the left and right sides of the roadside-marker post. The solar panel is mounted on the upper plane of the DSN.

## 3. Design of DSN Hardware

### 3.1. PIR Sensors

**Figure 5:**
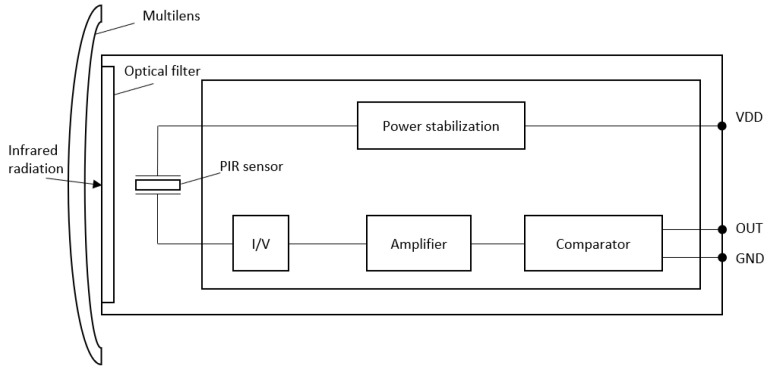
Internal structure of the PIR sensor [43].

Various sensors can be used for the potential detection of animals in the roadside area: light-beam interruption or reflection; radar; thermal radiation sensors; optical and/or thermal imaging; etc. Since it is required to cover relatively long stretches of road (hundreds of meters or more), the designed DSNs should be relatively cheap and with an autonomous power supply. For these reasons (cost and energy consumption), the use of video/thermal imaging sensors and radars has been abandoned. Since it was decided that DSNs will be installed on roadside-marker posts, which are placed on roadsides every 50 m or 100 m (on straight stretches of roadsides, the distance between the roadside-marker posts can technically and economically be increased to 100 m [40]), therefore, the distance of detection of the animal detection sensors should be at least 50 m. Pyroelectric (PIR) sensors that detect thermal infrared (IR) radiation (wavelength 8–14 μm) are the most energy efficient. PIR sensors have two or more thermal IR radiation passive pyroelectric sensitive elements. Pyroelectric sensors generate an electric charge only when the intensity of IR radiation changes, making them very suitable for detecting warm moving objects. These are among the least energy-consuming motion sensors. The current used by modern PIR sensors, together with the amplifier and the detection scheme (Figure 5), can be less than 30 µA [43,44].

To increase the detection distance (aperture) of the sensors, IR lenses are used for wavelengths from a few to tens of micrometers. To reduce cost and weight, plastic Fresnel multisegmented or simple lenses (sometimes mirrors or a combination of lenses and mirrors) are usually used.

Using PIR sensors with sector view multisegment lenses, and for potentially higher sensitivity (detection distance of moving warm objects), there is a high probability of false positives on a windy day when sun-heated tree or bush branches (tall grass) move near the sensors. Foggy nights, rainfall, and snowstorms can decrease the detection distance of the sensors. For this reason, it is more logical to concentrate on the directionality and detection distance of the sensors along the roadside.

In practice, PIR sensors with four different IR lenses were tested: one multisegment and three single-zone lenses from different manufacturers. The tests were carried out in the forest, when the air temperature was 20 °C (April 2021), and the objects—a person—were detected (see results in Table 2). Experiments were repeated with three different persons with weights of ~40 kg (object 1), ~60 kg (object 2), ~80 kg (object 3) and heights of 130 cm, 170 cm, and 190 cm. Each person walked on the forest path (average speed 5–6 km/h) from a distance of 60 m towards the PIR sensor. Then sensor-triggered measurements were taken. An average of 10 measurements with each object and each Fresnel lens is presented in Table 2. Here LENS 1 is a multisegmented Fresnel lens (Fresnel Factory Inc., San Jose, CA, USA, PF305-8324), LENS 2 (Fresnel Factory Inc. PF03-3025), LENS 3 (Fresnel Factory Inc. PF20-06015), and LENS 4 (Fresnel Factory Inc. PF28) are single-segment Fresnel lenses. The purpose of the tests is to determine, under equal conditions, which optical system will ensure the maximum detection distance. As expected, the maximum distance (nearly 40 m) was recorded by sensors with a single zone and the largest dimensions (LENS 3: d = 3 cm) Fresnel lens. The detection distance of the multisegment lens optical system was only 20 m. Even a detection distance of 40 m does not enable the length of the DSN sensitive zone to be increased to 100 m (this is the distance at which roadside-marker posts are now most often built in Lithuania on trunk and country roads), but it should be suitable if DSNs were installed every 50 m.

On country roads, the distance from the cleared roadside area to the trees, bushes, or tall grass is usually only a few meters, so the designed improved DSN prototypes include three PIR sensors: two aimed at a small angle from the road and the third with a small multisegment lens for monitoring the rear area of the DSN (detection distance up to 5 m).

PIR sensors react to a change in thermal radiation, which, in the case of a motion sensor, depends on the temperature difference between the detected object and the environment. If the temperature of the object and the environment are the same, the sensor will not be triggered. The authors photographed animals kept in enclosures with a thermal imaging camera, and it was confirmed that the recorded wavelengths are in a similar range to the PIR sensors.

### 3.2. Energy Consumption and Harvesting of the DSM Node

The main source of energy for DSNs is solar panels (photovoltaic cells). During the night, the devices will be powered by the battery.

The average current generated by solar panels throughout the day is:

*I_avg_* = (*T_d_* ∙ *I_sp_*)/24, (1)

where *T_d_* is the length of daylight (day) in hours and *I_sp_* is the average current generated by solar panels during the day.

The energy balance was estimated for the worst-case scenario, during a cloudy day on the shortest day. In December in Lithuania, the length of the day *T_d_* is only a little longer than 7 h, so in the following calculations *T_d_* = 7 h was accepted.

**Figure 6:**
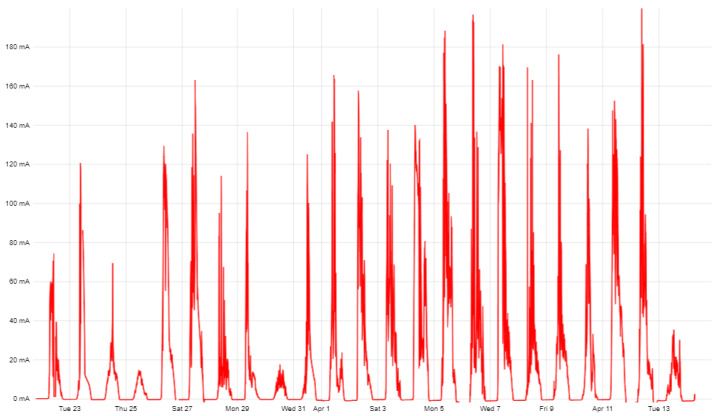
Charging current schedule from 22 March–13 April 2021. The average current generated during the entire period was *I_v_* = 16.8 mA.

The experimental assessment of the expected average solar panel current was performed under various lighting conditions in March–April 2021. The current generated by polycrystalline solar panels with an area of 66 cm^2^ (batteries of a similar size are expected to be used in DSN prototypes) was recorded when the voltage generated by it was higher than 3 V. The long-term data-acquisition results are shown in Figure 6.

**Figure 7:**
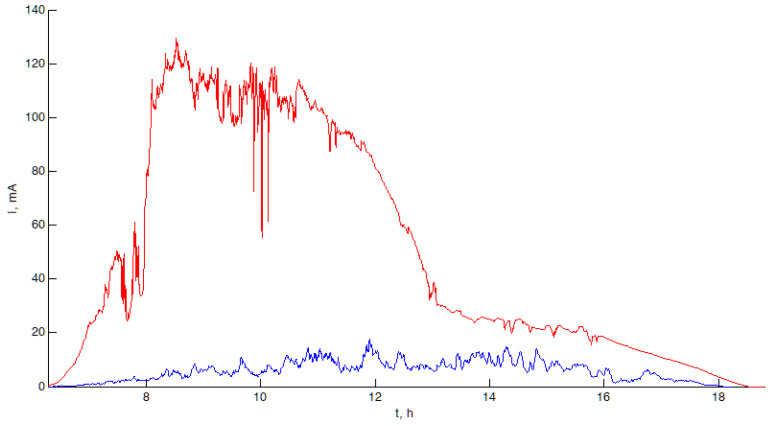
Charge currents generated by solar panels on 25 March 2021 (cloudy, rain, blue graph) and 26 March 2021 (sunny, red graph).

The average current *I_sp_* during daylight hours in cloudy and rainy weather is about 6 mA, while on a sunny day, it can reach 60 mA or more (depending on the orientation of the solar panel). The experimental results are shown in Figure 7.

When calculating the resources of the average generated current in the worst-case scenario, it was assumed that *I_vd_* = 6 mA. In December, under overcast weather, the average daily generated current will be only:

*I_v_* = (7 × 6)/24 = 1.75 mA (2)

The charge current can be even lower in the shade of forest trees, or when the surfaces of the solar panels are contaminated and obstructed. So, it was assumed that the current used by the DSN in standby mode, when only the most necessary node (sensors) is active, should not exceed 1 mA. Due to limited energy resources, it was decided not to use sensors based on the light-beam barrier in the prototypes, because their lights require a current of >20 mA.

The batteries are designed to power the DSN during the dark hours of the day. The majority of the batteries perform poorly at low temperatures—their capacity usually decreases and internal resistance increases. Since the DSN will have to work in a wide range of temperatures, it is necessary to choose suitable batteries that can be used properly in both high and low temperatures.

The capacity of lithium-ion batteries can decrease up to 55% at negative temperatures (<−20 °C) [45,46,47]. There are rechargeable cells on the market that do not degrade as much in the cold, but their price is high (~$25 [48]). Lithium-based batteries require specialized charging and discharging circuits to keep their voltages around 2.6...4.2 V. They can catch fire if overloaded or overcharged, therefore they often need additional protections to shut them down at temperatures higher than the manufacturer recommends.

NiMH batteries are safer than lithium (cannot self-ignite) and can be fully discharged and recharged (small current < 0.1 °C). The disadvantage of many NiMHs is their fairly significant self-discharge, but there are also NiMH batteries with an extremely low self-discharge current [49].

**Figure 8:**
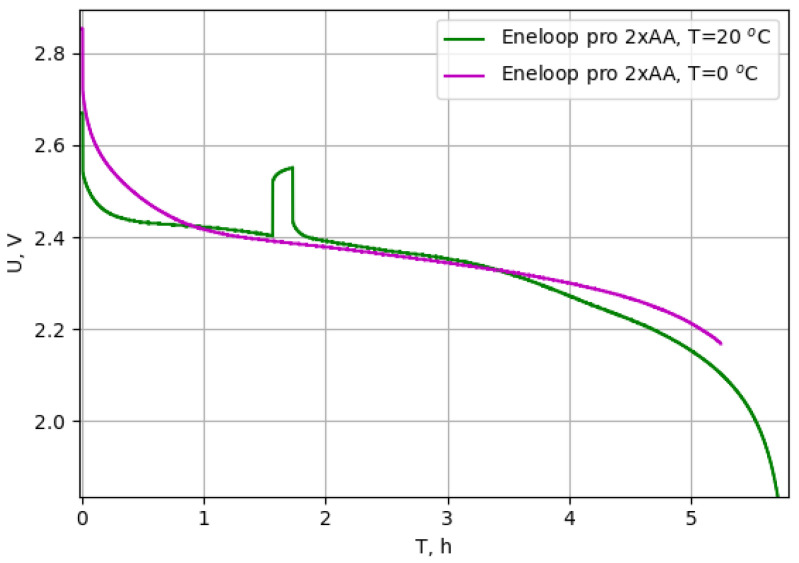
Panasonic Eneloop pro battery discharge test results at 20 °C and 0 °C. Voltage jump in the green graph is the moment of measuring the battery’s internal resistance and recovery parameters.

Since manufacturers rarely provide the parameters and characteristics of batteries when they work at low temperatures, several lithium-ion (Panasonic (Osaka, Japan) and Samsung (Suwon, Republic of Korea) 18650 type) and nickel-metal hydrate batteries (Panasonic Eneloop pro) were tested. For Panasonic Eneloop batteries, their capacity practically did not change at temperatures of 20 °C and 0 °C. Figure 8 shows the test results of these batteries (connected two in a series) with a current of 400 mA at different temperatures.

In the DSN prototypes, it was decided to use three AA size 2450 mAh Panasonic Eneloop pro batteries due to their safety (compared to Li-ion batteries) and capacity stability over a wide range of temperatures. The discharge curve is given in Figure 8.

The block diagram in Figure 2 shows the components of the DSN. The energy costs of the DSN node will be the sum of the energy costs of the individual elements, which can be expressed by the formula:

*I_SUM_* = 3 × *I_PIR_* + *I_RF_* + *I_MCU_* + 2 × *I_LED_* (3)

The DSN node will spend most of its time in sleep mode when its energy consumption will be the lowest. According to the data presented in the manufacturer’s documentation [42,43,44,50], the energy consumption of the DSN node will be:

*I_SUM_sleep_ =* 3 × 20 μA + 1 μA + 10 μA + 2 × 0 μA = 71 μA (4)

In active mode, the DSN node will consume:

*I_SUM_active_* = 3 × 20 μA + 20 mA + 2 mA + 2 × 50 mA = 122.6 mA (5)

It is likely that the appearance of wild animals on the road section during the day will not be very frequent, but false alarms due to the movement of other warm objects are also possible.

DSN is the expected periodic wake-up of the processor and short-term activation of the receiving part of the radio communication module (to detect the activation of other DSNs). This will also slightly increase the standby current. In any case, in the absence of many sensor activations during the day, it is very likely that the average current consumed by the DSN electronics will be less than 1 mA. Even assuming that the average DSN current consumption will be 1 mA, the module should last over three months from a fully charged battery (2450 mAh).

## 4. Experiments

**Figure 9:**
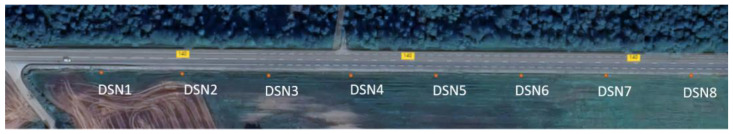
DSN installation positions near the road.

For experimental research, the first batch of DSNs (eight units) was produced with slightly different angles of placement of the sensors in relation to the road (inclination of the side sensors from the road −5 and −15 degrees) and also with a rain cover installed over one of the side sensors. Sensors were installed on the Kaunas–Šakiai (41 km) road section (Figure 9) for one month.

A photo camera was installed next to the DSN8 sensor. It is triggered by sensor activations. A wireless network is formed between the sensors and the camera using LoRa technology. When at least one of the sensors is triggered, the camera captures three photos at 200 ms intervals. Photos are stored in the camera (SD card). The camera provides an opportunity to view low-resolution photocopies that are placed in the camera manufacturer’s database. To collect statistics and monitor the current situation in real time, a gateway based on Rpi4 was made. The gateway is connected to the sensor network using the LoRa technology, and a 4G modem was used for real-time monitoring. It was possible to connect to the gateway remotely and download the accumulated database. Physically, the gateway is placed 500 m away from the road section on the premises of the Sutkai Forestry. The first batches of DSNs were tested from July to October 2022.

**Figure 10:**
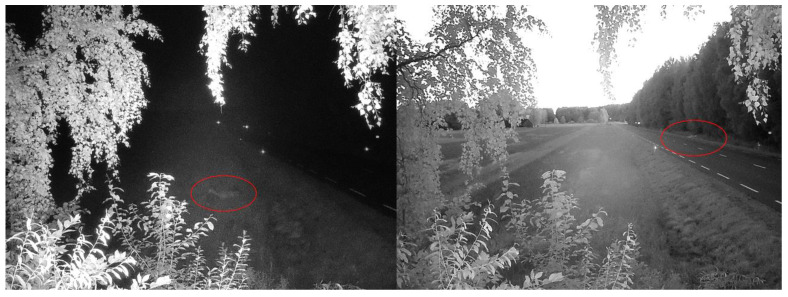
Photos of the detected wildlife.

During the first weeks of monitoring, 10 sensor activations on average were recorded during the dark period of the day. Analyzing the camera images, several captured animals were found. Example photos (evening/night) are shown in Figure 10.

From the provided sample photos, we can see that, in the dark, the camera allows for capturing objects up to 30 m away from its installation location. Therefore, the verification of the reasons for the activation of DSN sensors becomes problematic.

It was observed that most of the triggers were caused by the rear PIR sensor rather than the side ones. The most likely reason for this could have been grass, tree, and shrub branches moved by the wind. Since only a small area of the roadside is maintained (mowing, snow plowing), and the forest is only a few meters away from the roadside-marker post, it was decided to abandon this sensor, providing only the option for the installation of the rear PIR sensor unit in the following DSN versions.

As expected, the sensors’ work was also affected by environmental conditions: rain, contamination of the sensors’ optics and taller grassy plants growing near them.

**Figure 11:**
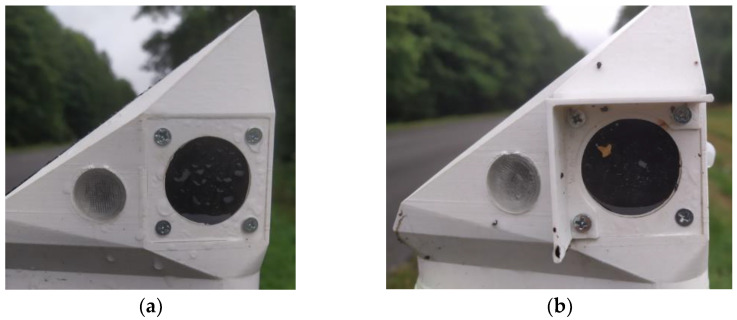
Photos of the installed first generation of DSN nodes with various types of optical contamination: (**a**) Raindrops on the sensor opening; (**b**) Organic particles and raindrops on the sensor opening with the installed canopy.

It was observed that the DSN with a canopy over the PIR sensor optics, contrary to expectations, got significantly dirtier (dirt is not easily washed away by rain: see Figure 11).

**Figure 12:**
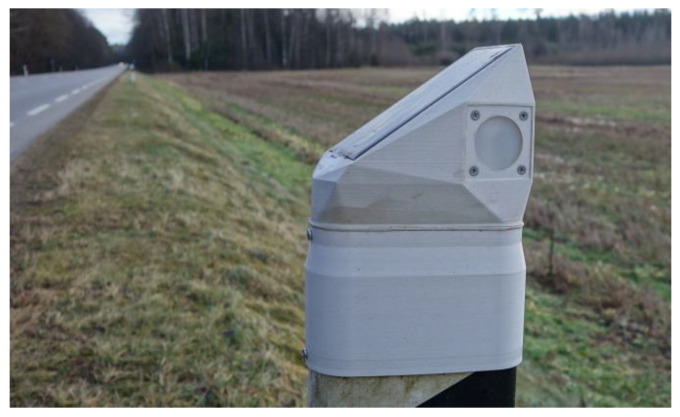
The second-generation DSN mounted on a roadside-marker post.

After the tests of the first DSN generation, it was decided to modify the detection and signaling units to some extent:

- The rear PIR sensor was removed, leaving only the possibility of additional installation of a separate submodule;
- The finally selected optical angle of the side sensors is equal to −5 degrees in relation to the road line. Such an angle allows you to avoid the detection of passing cars and does not deviate too far from the side of the road;
- The Fresnel lens of the side PIR sensors has been changed from black to white (the entire DSN remains white: see Figure 12);
- Red LED signal indicators are aligned with the PIR optics unit, where the Fresnel lens also performs the function of an optical signaling window. Tests have confirmed that LED flashing in the mod does not trigger the PIR sensor;
- The photoresistor for determining the dark time of the day is abandoned, using a modified solar battery voltage measurement circuit solution for this purpose;
- Two options for installing the DSN on the roadside-marker post were proposed: by cutting off its upper part (visually more solid) or by screwing it on and fixing it on the upper part of the unmodified roadside-marker post.

It was decided to use a thermal imaging camera (Hikvision DS-2TD2617-6/PA). The thermal images taken for visual reference are of relatively low resolution (160 × 120) for identification of the presence of wildlife and classification of the source of the triggering instead of a hunting camera with night illumination to check the reasons for the activation of the second generation DSN sensors (Figure 12). The IoT network changed after starting to use the second generation of DSN sensors. LoRa gateway stores triggering data received from the DSN sensors. The same gateway is used to control and store data received from the thermal camera. Both data sets’ pictures and triggering logs are sent to the cloud server using a 4G modem once per day. It is possible to configure the LoRa gateway to send all the data (photos and logs) immediately, but, at the experiment site, the 4G network is unstable. Therefore, it was decided to store all data locally and send it to the cloud periodically.

It was decided to use a thermal imaging camera instead of a hunting camera with night illumination to check the reasons for the activation of the second series ASM sensors. After analyzing the thermal imaging cameras available on the market, the original idea of using a battery-powered camera was rejected due to the relatively short operating time of the camera (depending on the model, lasting from a few to a dozen hours).

After consultation with the customer and after obtaining permission from the Road Directorate, it was decided to install a thermal imaging camera (160 × 120 pixels) fed from the network. Photo cameras used near the Road Directorate on a roadside-marker post are located at km 41. The data-collecting device with a camera was installed on the roadside-marker post. The thermal imaging camera can record both photos and thermal imaging images. For data-protection purposes, only the thermal images are recorded. The data acquisition during the system research duration is continuous (24/7). The photographing and data-saving period of the data-acquisition system was set to 10 s. The selected setup and sampling period allows identification of the false activations of DSN sensors in those cases when the DSN sensor was activated, but no wild animals were photographed in the investigated area. These cases, when an animal was photographed in the investigated area, but the sensors were not activated, can be identified as well.

Tests of the second-generation DSN with modified data registration and verification nodes with a thermal imaging camera were installed and tested from October 2021 to February 2022.

On 4 December 2021, the sensors stopped sending signals. After checking, it was noticed that their fastening part was broken, and the DSN was lying on the side of the road. While observing the snow-clearing works on the road, it was noticed that the clearing truck shook the roadside-marker posts quite strongly while clearing the snow. In the case of negative temperatures, this could have been the reason for the breakdown of the fasteners.

It was also noticed that one of the PIR sensors of one DSN was turning on significantly more often than other DSNs. It was found that it was reacting to passing cars. After going to the installation site, it turned out that the roadside-marker post on which this DSN was installed was slightly turned in relation to the road, which had the effect that one of the sensors covered a segment of the road rather than the sidewalk. This imposes certain requirements, although not as strict as in the case of the light-beam barrier variant, for the accuracy of the DSN mounting on the roadside-marker post. There are also possible problems with car detection in turn segments of the road. In this case, the modified DSNs should be installed (with different angles of sensor optical nodes), or sensors that are tilted towards the roadway should be disconnected.

**Figure 13:**
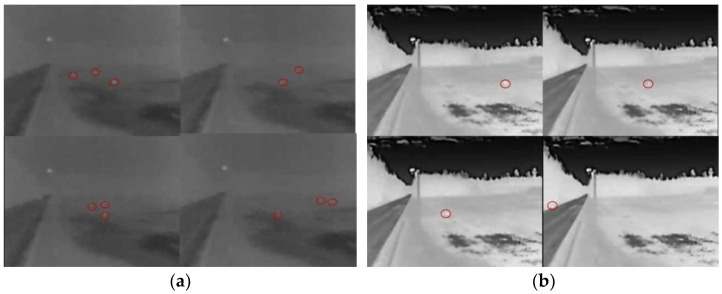
Thermovision photos of the detected wildlife: (**a**) The group of animals crossing the road from left to right; (**b**) One animal crossing the road from right to left.

From the provided photos (Figure 13a), it can be seen that three objects are moving from the road (dark area on the left side of the photo) towards the fields. The DSN1 and DSN2 sensors recorded objects near the road. The photos in Figure 13b also show one captured animal moving from the right side (from the field) to the left (towards the road) and crossing the road. In this case, the DSN1 sensor detected animals.

**Figure 14:**
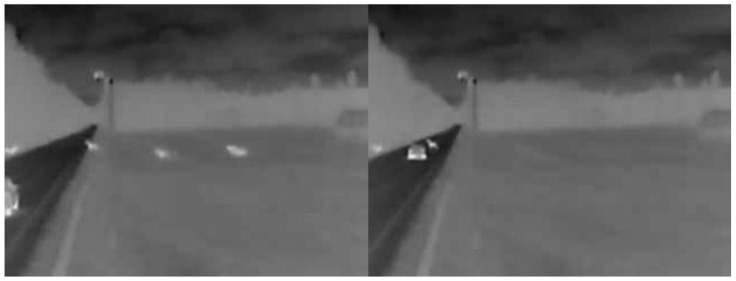
A series of thermal imaging images captured on 6 April at 5 a.m. 54 min, indicating a time-recorded dangerous incident.

After extending the research in March and April 2022, as expected, a significant increase in animal activity was observed in the monitored section (especially in April). On 6 April at 5:00 a.m. 54 min, a series of thermal imaging images of a timed emergency were captured (Figure 14), where a group of several deer ran across the road from the right side, and the last animal was nearly hit by a passing car.

DSN nodes in the experimental setup start working after dark till early morning. At different times of the year, different wildlife activities are seen. For example, on 14 November 2021, four activations per day were recorded. After analyzing the thermal images, a small animal (a cat or small dog) is seen moving in the field next to the road in the sensitivity zone of the system, resulting in three DSN triggers. One DSN triggering can be classified as false. This gives us 25 percent false DSN triggering. On the other hand, after one week, we had 24 DSN triggers. After analysis of the thermal photos, we identified a group of three (to the best of the authors’ knowledge from the acquired data) deer walking back and forth across the field for several hours. In all cases of DSN triggering, it can be said that the wild animals were within the DSN coverage field. This gives us zero percent false positive DSN triggers. The required network coverage in the experimental setup of the research is within 1 km.; the DSN nodes are in a direct line of sight which makes a LoRa link virtually uninterrupted, and the packet-reception ratio in the experiment was close to 100%. The redundant repeating of data packets three times was applied in the system-communication algorithm for lost packet control. An analysis of the data taken during one month shows that false triggering of the DSN nodes is 2–3%.

## 5. Discussion and Conclusions

During the trial of the system, which lasted from July 2021 to April 2022, the trial operation of the DSN nodes and the registration node was carried out in the designated road section.

During the first months of testing, several different DSN design options were tested: several different mounting angles of the side PIR sensor lenses and the impact of the rain canopy.

After analyzing the performance of the first-generation DSN sensors, the design of the second-generation DSN was modified: a combined design of side PIR sensors and an LED signaling node with white Fresnel lenses was selected; the rear PIR sensor was removed since its activations both near the forest and near the meadow/field are more misleading due to impact of plants, bushes, and branches moving in the wind.

A modified unit for registration and verification of DSN triggers was proposed and implemented. The first version with a hunter camera responding to DSN triggers and photographing in optical range with short-wave infrared illumination was rejected due to the covered distance being too small. It was replaced with a thermal imaging camera. The registration unit itself has been moved to a roadside-marker post. This allowed for a much more reliable assessment of the activations of the installed DSNs in the viewing area of this node reaching up to 300 m.

During the tests, even at the darkest time of the year, lasting from November to January, the autonomous power supply of the DSN from the integrated solar panels and batteries was sufficient. In an untypical situation due to a statistically excessive number of sensor activations or drained battery, DSN nodes could switch to safe-operation mode, where the main priority is energy saving/accumulation in the battery up to a level suitable for operating.

It was found that the DSN practically always reacted to the larger animals (e.g., deer) running into the monitored roadside area.

The main causes of false DSN sensor triggers were those caused by nonanimals. They were due to passing large trucks and buses, most likely driving on wet road surfaces.

Single and hard-to-explain, repeating less than a few times per day, activations were mostly observed in foggy conditions and sometimes during rain and snowfall. In the warm season, grassy plants growing near the DSN sensors and arachnids moving in the sensor aperture area can also cause false activations.

Analysis of the IR images from April 2022, when many animals were recorded each night, showed that, on some nights, the animals stayed in the meadow next to the road for quite a long time, lasting up to several hours. In these cases, there may be a problem with warning the drivers about them through the DSN light signal because of excessive energy consumption from the autonomous DSN battery. The monitoring system proposed in the paper has a LoRa gateway which collects data from the DSN nodes. The same gateway is connected to the IoT network using a 4G modem. All collected data can be sent/processed via cloud services. End users who will approach near the monitoring area can be informed via a mobile app that animals were spotted nearby.

## Figures and Tables

**Table 1:** LPWAN technology parameters.

Parameter	LoRaWAN	Sigfox	NB-IoT
Range	<5 km (Urban), <15 km (Rural)	<10 km (Urban), <50 km (Rural)	<15 km
Spectrum	Sub-GHz ISM: EU (433, 868 MHz) US (915 MHz)	Sub-GHz ISM: EU (868 MHz) US (902 MHz)	Licensed (700–900 MHz)
Bandwidth	125–500 kHz	100 Hz	180 kHz
Data Rate	0.3–37.5 kbps (LoRa) 50 kbps (FSK)	<100 bps (uplink) <600 bps (downlink)	<150 kbps

**Table 2:** Experimental results of lens-diameter influence on object-detection distance.

Lens Dimensions	Object 1 Average Distance, m	Object 2 Average Distance, m	Object 3 Average Distance, m
LENS 1 (d = 25 mm)	19.5	20.5	21.1
LENS 2 (d = 16 mm)	35.6	36.2	36.8
LENS 3 (d = 29 mm)	40.2	41.3	41.1
LENS 4 (d = 27 mm)	37.8	39.1	39.3

## Data Availability

Not applicable.
